# Deep Learning on Construction Sites: A Case Study of Sparse Data Learning Techniques for Rebar Segmentation

**DOI:** 10.3390/s21165428

**Published:** 2021-08-11

**Authors:** Suzanna Cuypers, Maarten Bassier, Maarten Vergauwen

**Affiliations:** Department of Civil Engineering, Geomatics Section, KU Leuven—Faculty of Engineering Technology, 9000 Ghent, Belgium; maarten.bassier@kuleuven.be (M.B.); maarten.vergauwen@kuleuven.be (M.V.)

**Keywords:** weakly-supervised learning, semi-supervised learning, image segmentation, remote sensing, construction site monitoring, cross-consistency training

## Abstract

Recent advances in deep learning models for image interpretation finally made it possible to automate construction site monitoring processes that rely on remote sensing. However, the major drawback of these models is their dependency on large datasets of training images labeled at pixel level, which must be produced manually by skilled personnel. To reduce the need for training data, this study evaluates weakly and semi-supervised semantic segmentation models for construction site imagery to efficiently automate monitoring tasks. As a case study, we compare fully, weakly and semi-supervised methods for the detection of rebar covers, which are useful for quality control. In the experiments, recent models, i.e., IRNet, DeepLabv3+ and the cross-consistency training model are compared for their ability to segment rebar covers from construction site imagery with minimal manual input. The results show that weakly and semi-supervised models can indeed rival with the performance of fully supervised models with the majority of the target objects being properly found. This study provides construction site stakeholders with detailed information on how to leverage deep learning for efficient construction site monitoring and weigh preprocessing, training, and testing efforts against each other in order to decide between fully, weakly and semi-supervised training.

## 1. Introduction

The automation of construction site monitoring is long overdue, especially for progress monitoring, quality inspections and quantity take-offs. These processes rely on visual inputs of either workers or construction site footage and are essential to ensure progression, quality, safety and productivity on site [1,2,3]. Currently, the inspections themselves and the analysis of the footage are performed manually, which is labor intensive and requires highly skilled personnel. As a result, only a subset of the site is inspected, leading to high failure costs on construction projects [4].

Deep learning methods for image interpretation offer a promising solution. Recent advancements in convolutional neural networks (CNNs) allow for unprecedented class and instance segmentation rates even in the most challenging conditions [5,6]. These deep learning models can be trained with nearly any goal function and inherently offer more holistic solutions than heuristic algorithms. Furthermore, they are fast and low-cost solutions that are also precise and more objective than manual inspection techniques [2]. However, a major bottleneck of deep learning is in the manual pixel labeling of the thousands of images that are needed to train the network parameters. Additionally, the imagery is often non-ideal due to varying weather conditions, clutter and construction operations. This further negatively impacts the amount of training data needed to generalize the models. Recent developments explore the incorporation of weakly-labeled data and even unlabeled data to overcome the training obstacles. However, these methods are currently unexplored for construction site monitoring.

**Figure 1:**
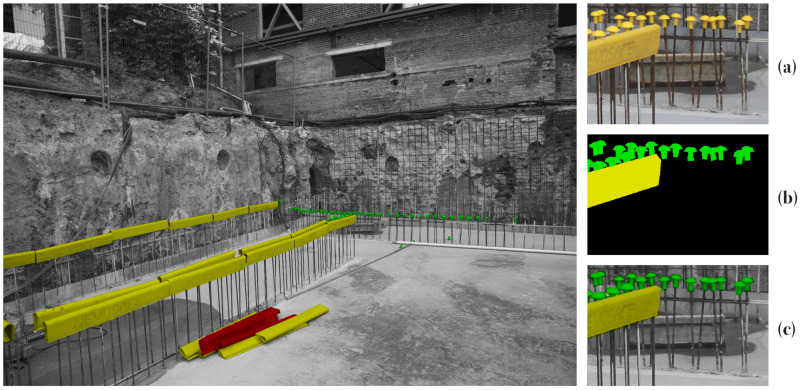
Segmentation for the detection of rebar covers for quality control on a 3744×5616 gray-scale image with segmented objects in color: (**a**) Detailed call-out of the original image, (**b**) ground-truth semantic segmentation labels and (**c**) overlay of the ground-truth labels on a black-and-white input image.

The goal of this work is to investigate how deep learning models can be efficiently adapted for construction site monitoring tasks. More specifically, we adapt state-of-the-art labeling and training methods to establish deep learning models to segment objects from construction site imagery. As a case study, we investigate the segmentation of different types of rebar covers of columns and walls (Figure 1). This is an essential validation task that is labor intensive but can avoid major failure costs [7]. Furthermore, it is a straightforward application with clear class delineations that should yield high detection rates with conventional neural networks. As such, the main contributions of this study are as follows:

1. A comparison of three training methods for rebar segmentation: fully-supervised learning with DeepLabv3+, weakly-supervised learning using IRNet and semi-supervised learning with cross-consistency training (CCT).
2. Empirical and visual performance analysis of the three models in realistic conditions.
3. The implementation of a new loss function in CCT to solve the class-imbalance problem caused by the low pixel presence of small objects such as rebar covers.

The remainder of this paper is structured as follows. First, the related work on deep learning adaptation for construction applications is discussed in Section 2. Next, the network architectures of state-of-the-art CNNs and learning methods are discussed in Section 3. In Section 4, we adapt three promising learning methods for the segmentation of rebar covers. The datasets are presented in Section 5. In Section 6 and Section 7, the three methods are compared and their performances discussed. Finally, the conclusions are presented in Section 8 along with insights for construction stakeholders who wish to apply machine learning to construction site imagery with limited training data available.

## 2. Related Work

The adaptation of machine learning and computer vision techniques is currently a hot topic in construction. These techniques are being developed for the purpose of safety management, damage detection, monitoring and BIM. The overview paper of Xu et al. [8] (2021) provides an in-depth literature review and history of the application of deep learning in construction. Similarly, Koch et al. [9] describe several traditional visual inspection methods and computer vision methods for civil engineering applications. Due to the costly and unreliable nature of manual inspections, both works stress the need for computer vision and deep learning methods to be applied to construction. To our knowledge, we are the first to segment rebar covers from construction site imagery. As such, the related work section of this paper focuses on works that explore similar techniques to automate construction monitoring, the comparison of unsupervised techniques and technologies to reduce training data requirements.

Of particular interest to this research is the comparison between the different automation techniques. A popular application is the detection of workers’ hardhats to evaluate safety compliance on site. Mneymneh et al. [10] compare three non-machine learning methods for this detection, i.e., feature matching, template matching and a cascade classifier with histogram of oriented gradients (HOG) features. They conclude that the accuracy of conventional computer vision methods is insufficient to deal with construction site imagery. In contrast, Wang et al. [1] use a CNN to detect hardhats in images using bounding boxes. Their network is trained on a self-collected and self-labeled dataset and achieves high average precision for people with and without hardhats. Similarly, Son et al. [3] apply a CNN for safety management and productivity analysis by detecting construction workers on site. They train Faster R-CNN fully supervised to detect workers in various poses and conditions, achieving very high accuracy. They enhance their datasets with ImageNet [11] and Microsoft COCO 2014 [12], showing that large public datasets can in fact be used to lower training data requirements for construction site applications. Wei et al. [13] also design a network to enhance safety on construction sites. They utilize a CNN method to detect workers performing unsafe actions and their identity in videos. An interesting aspect of their method is that they implement a spatial and temporal attention pooling network to filter redundant information from the images. They show that deep learning methods can perform near real-time with high detection rates if properly trained.

A second application that is often compared in the literature is damage detection. For instance, Manjurul Islam et al. [2] use an encoder–decoder network to segment cracks in concrete structures. Their network contains the classification network VGG16 as a backbone network and is trained fully supervised on a benchmark dataset containing 40,000 images for crack classification [14]. Their work shows that multiple public datasets can be combined for new applications. Dung et al. [15] use the same dataset and train three different classification networks fully supervised to detect cracks: VGG16, Inception, and ResNet-152. Furthermore, they manually annotate only 600 images of that dataset to create a network from limited additional training data. They conclude that, although their method achieves a reasonable quality of detection, further work is required to make crack detection more robust against noise. Deng et al. [16] also opt for a minimal training data approach and employ an atrous spatial pyramid pooling module and include a weight balanced intersection over union (IoU) loss function to mediate the class-imbalance in the training data. A promising work with the same goal is from Guo et al. [17], who describe a semi-supervised method to classify defects on facades. They incorporate an uncertainty filter to select reliable unlabeled data to improve the network’s prediction from limited data. Their method, while it is able to classify the defects in a given image, cannot detect or segment the defective area. Our work focuses on the exact localization of the objects in the image through image segmentation, which is considered a step up from classification.

## 3. Deep Learning Methods

As discussed above, deep learning models can outperform conventional methods given a representative and balanced training dataset. With regard to the availability of training data, there currently are four main categories of learning methods relevant to this work: fully-supervised, weakly-supervised, semi-supervised and zero-shot learning. In this paper, only the first three are discussed since zero-shot learning with very few or no training data is currently very much a subject of fundamental research that is still far from being directly applied to construction site applications. This section reviews the first three categories as well as it specifically explains the three networks that will be adapted for the rebar segmentation, i.e., Deeplabv3+, IRNet and cross-consistency training (CCT).

### 3.1. Fully-Supervised Learning

The most common way to train a segmentation CNN is fully supervised by giving the network fully labeled samples from which it can learn. To enhance the performance of the model, data augmentation techniques can be applied to the training data and a pretrained network can be transferred to the specific image domain. U-Net [18], SegNet [19,20], DeepLab [21,22,23], FPN [24] and PSPNet [25] are commonly used segmentation models that achieve state-of-the-art performance. Such models generally consist of an encoder and a decoder. The encoder is a classification network that extracts rich semantic information from the image, and the decoder recovers localization information and sharp object boundaries. In this work, we use the DeepLab encoder–decoder network by Chen et al. [21], of which the latest one is DeepLabv3+ [23]. By using atrous convolution in its encoder, features are extracted at different scales. The encoder module is based on the classification network ResNet or Xception. In the case of Xception, the authors employ depthwise separable convolution, which improves the speed and accuracy.

To expand the dataset without generating more labeled samples, several augmentation functions can be applied to the data such as cropping, vertical and horizontal flipping, rotating and scaling [26,27]. In [28], van Noord et al. explain that data augmentation such as scaling does not only expand the training set, but also reduces overfitting because the model is trained to recognize the features both in the dataset and at a different scale, thus making the model scale-invariant.

For most applications with CNNs, transfer learning is applied. This means that the network is first trained on a larger public image dataset such as ImageNet [11] and then the learned knowledge of the net is transferred to the specific domain of the application. In the pretraining step, the net initializes its weights. It learns to understand basic image concepts such as color and edges in the first layers of the net followed by shape and texture [26]. The actual recognizing of the object class happens in the last layers of the network. When a net is already trained to detect objects from large public datasets, it can quickly adapt and learn to detect other objects. When training on the intended dataset, the initial layers of the net are typically fixed so that only the last layers in the net will be updated and fine-tuned. As a result, the net can learn from small datasets and still perform well [29].

### 3.2. Weakly-Supervised Learning

Weakly-supervised learning makes use of lower-level annotations than the intended output of the network, e.g., using image classifications to reinforce pixel-segmentations. Most weakly-supervised learning advancements explore the option of using image-level labels for image segmentation [6,30,31,32,33]. These labels can be used to acquire strong localization cues for semantic segmentation. In this study, we employ image-level tags as a supervision method.

A commonly used method to extract localization information from image-level labels is through Class Activation Maps (CAMs). A CAM is the weighted sum of the outputs of the global average pooling layer, at the last convolutional layer in the network. The weights stem from the fully connected layer, which is linked to all the classes. These weights determine which units of the last convolutional layer have the largest impact on the prediction of the class. This is used in weakly-supervised learning as a localization method and is obtained from classification networks [34]. While a network trained for classification does not provide the exact location of the object in the final layer, it does look at specific locations in the image to make its decision. For each class, a CAM can be obtained. This provides approximate locations of various objects in the image.

From CAMs we can learn which elements of the object are used for the discrimination of the class [35]. For example, to detect a specific dog breed, the unit that looks at the dog’s face and the unit that focuses on the body and fur are combined. Moreover, there are several benefits of using CAMs to visualize the most important regions in the image that determine the net’s decision. CAMs help identify failure models and create trust and confidence in the use of the net [36].

Much research has been done on weak supervision by means of CAMs [33,34,36,37,38]. However, as Ahn et al. [32] mention, CAMs cannot serve as labels for the training samples due to their limited resolution and inability to distinguish instances and define accurate boundaries. The authors amend the drawbacks of CAMs by using an inter-pixel relation network, called IRNet. We employ their framework in our experiments.

The IRNet framework trains a weakly-supervised pseudo-instance segmentation label generator from image-level labels. As such, the net learns from weakly-labeled images, but produces pixel-level labels. These are pseudo-labels that can be used to train another segmentation network fully supervised. IRNet in itself cannot be used to perform segmentation on images without given image-level labels. Therefore, the outputs are called pseudo-labels.

IRNet uses CAMs to define seed areas and then propagates these to cover the entire object area. Additionally, the CAMs also serve as a supervision for IRNet by extracting inter-pixel relations. IRNet is a two-branch network that generates a displacement vector field and a class boundary map. The displacement vector field, generated by the first branch, is a map that contains a 2D vector at each pixel location pointing to the centroid of the instance the pixel belongs to. This displacement field is then used to generate a class-agnostic instance map in which each pixel that belongs to the same centroid is given the same instance label. Class boundary maps are generated by the second branch of IRNet. Using the boundaries between different object classes, the pairwise semantic affinities can be retrieved. If two pixels are separated by a strong object boundary, their semantic affinity will be low. Thus, a high semantic affinity score indicates a high confidence score for belonging to the same class. The instance map generated by the first branch of IRNet can be combined with CAMs. The CAMs, extracted from a classification network, contain the class information, whereas the instance maps contain the instance information. Combining the CAMs with the instance maps results in instance-wise CAMs. The boundaries of these CAMs are refined using the pairwise affinities resulting from the second branch. The framework can either output instance or semantic segmentation labels. The pseudo-instance or semantic segmentation labels can then be used to train a segmentation network fully supervised.

### 3.3. Semi-Supervised Learning

The third and final learning method is semi-supervised learning. In semi-supervised learning, a net is trained with a small set of labeled images and a large set of unlabeled data. Although creating just a few fully-labeled samples does not require too much work, manually labeling data is a laborsome task. Therefore, semi-supervised learning has drawn a lot of attention in recent works [5,39,40]. There are various approaches to semi-supervised learning such as generative adversarial networks [40,41,42], teacher–student models [43] and universal learning [44].

For semantic segmentation, most semi-supervised methods use generative adversarial networks (GANs) [40,41,42]. A GAN framework consists of two parts: a generator and a discriminator. The former creates additional training samples using noise, the latter discriminates the fake training pixel samples from the real samples by assigning them a class label or marking them as fake [41].

In this study we employ CCT as proposed by Ouali et al. [5]. It is a consistency-based semi-supervised semantic segmentation method that eliminates the two drawbacks of weakly- and semi-supervised learning methods. The first drawback is that weakly-supervised methods do not exploit unlabeled data, and the second is that semi-supervised methods, although they use the unlabeled data, are a lot harder to train. The CCT model is a simple framework that exploits the unlabeled samples by enforcing consistency of the prediction for perturbed images. The various perturbations are applied on the hidden representation level of the input image. CCT makes the model robust to small changes. As a result, the encoder part of the model is further optimized, coming at a small computational and memory cost. Consistency training is highly dependent on the data distribution due to the cluster assumption, which dictates that all classes must be separated by low-density regions. By measuring the local variations between each pixel and its neighbors, the local smoothness is estimated to confirm the cluster assumption. This is done by calculating the average Euclidean distance at each spatial location and its eight intermediate neighboring pixels for both the inputs and the upsampled hidden representations (2048-dimensional feature maps), which are generated by a DeepLabv3 ResNet encoder [22]. Given that, if experimentally proven, the cluster assumption is not met at the input level, the perturbations are applied to the encoder’s outputs only.

The proposed framework consists of an encoder and multiple decoders. The shared encoder and main decoder are first trained on the small labeled dataset. Then, the result of the shared encoder and main decoder on an unlabeled image is compared to the result generated by a set of auxiliary decoders that have been fed perturbations of the encoded feature representations. Forcing a consistency in the predictions between the result of the main decoder and the auxiliary decoders through a loss function enhances the performance of the shared encoder and, therefore, the whole segmentation network.

A drawback of the CCT framework is that it is designed to employ the same images in the weakly-labeled dataset as in the unlabeled dataset. Moreover, the CCT model contains batch normalization [45], which means that the mean and standard deviation are calculated over a mini-batch of training samples and not the entire dataset. This speeds up the training, but should be avoided if the two samples are not representative of the classes. Since our images rarely contain all three objects and many (in $D_{l}$) only contain background, this might cause a poorly fitting model.

## 4. Methods

In this study, we adapt two recent learning frameworks: the weakly-supervised IRNet and the semi-supervised cross-consistency training. To compare the performance of the two frameworks, we also train two fully-supervised DeepLabv3+ models and use these as a baseline. In the following subsections, we describe how we train IRNet, CCT and the two Deeplabv3+ reference models. For the training, we used a PC with an Intel Xeon W-2133 processor, 32 GB of RAM and an NVIDIA GeForce GTX 1080 GPU running Ubuntu 16.04. IRNet and CCT were trained on Linux using a Python codebase while the two Deeplabv3+ models were trained using Matlab on an identical computer running Windows.

### 4.1. Training Setup of IRNet

**Figure 2:**
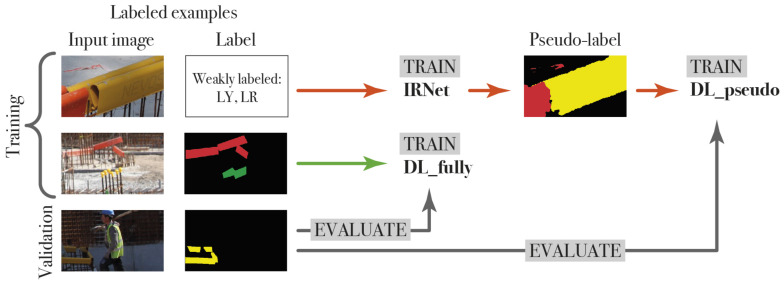
IRNet and DL_fully (fully-supervised Deeplabv3+) are trained using the training set. DL_pseudo is trained with the pseudo-labels generated by IRNet. The testing images are used to evaluate IRNet, DL_pseudo and DL_fully.

IRNet is trained on a training set that contains 1065 images. To fit the capacity of the GPU, the batch size is reduced to 8. The testing set is used to evaluate the quality of the pseudo-labels. For the entire dataset, pseudo-labels are generated with which a DeepLabv3+ ResNet-18 model is trained, called DL_pseudo. Ahn et al. [32] also trained a Deeplabv3+ model with pseudo-labels and compared their method to a fully-supervised Deeplabv3+ model. The performance of DL_pseudo was evaluated on the testing set. To compare the weakly-supervised training method of IRNet, the same DeepLabv3+ model was also trained fully supervised on the training set and evaluated on the testing set. We call this model DL_fully and will use it as a reference. Figure 2 shows our training setup.

DeepLabv3+ ResNet18 is trained with 5 different option settings (initial learning rate of 0.01, 0.003, 0.001, 0.0003, 0.0001) and a batch size of 8; the net with the best accuracy and loss is chosen. For DL_fully the best result is trained with an initial learning rate of 0.01. DL_pseudo is trained with an initial learning rate of 0.001.

### 4.2. Training Setup of CCT

The CCT model is a semi-supervised model that can be expanded to also make use of weakly-labeled data, making it simultaneously a weakly- and semi-supervised method. Before explaining the various tested models, we first go over some training options that are identical for all the tests. We explain the chosen perturbation functions. Then, we expand the model by applying different loss functions. Finally, we display the various supervision methods and list their datasets. For other training parameters, we use the same ones as the authors [5], namely an initial learning rate of 0.02, a weight decay of 0.0004 and a momentum of 0.9. The batch size is also set at 10.

#### 4.2.1. Perturbation Functions

Ouali et al. [5] apply perturbations in their CCT framework to modify the output of the encoder network before feeding it to auxiliary decoder networks. The predictions by the auxiliary decoders are then compared to the output of the main decoder, which is trained fully supervised. The unsupervised loss is computed between the outputs of the auxiliary decoders and the main decoder. The total loss combining the supervised and weighted unsupervised loss is used to train the shared encoder and auxiliary decoders.

Since the authors found that there is an insignificant performance difference between different perturbation functions and that a combination of all perturbations gives an additional small improvement in the performance, we use the same setting they used in the rest of their experiments: 2 auxiliary decoders for each of the perturbation functions Con-Msk, Obj-Msk and I-VAT, as well as 6 auxiliary decoders for each of the perturbation functions G-Cutout, F-drop, F-noise and DropOut.

#### 4.2.2. Loss Functions

Several loss functions are integrated in the CCT model. The model employs a different loss for the fully-supervised branch, the unsupervised branch and the weakly-supervised branch. In this study, we test three different functions for the fully-supervised loss component. We use cross-entropy (**CE**) loss (Equation (1)), an annealed bootstrapped cross-entropy (ab-**CE**) loss (Equation (2)) and focal loss (**FL**) [46], which was also employed by the authors:

(1) $$L_{s}=\frac{1}{|D_{l}|}\sum_{y\in D_{l}}\mathrm{CE}\left(y,p\right)$$

(2) $$L_{s}=\frac{1}{|D_{l}|}\sum_{y\in D_{l}}\left\{f\left(x\right)<\eta \right\}\mathrm{CE}\left(y,p\right)$$

where $|D_{l}|$ is the number of elements (we take the average of all losses of the batch size) and *y* represents the ground truth. The probability distribution $p\in R^{C\times H\times W}$ is the prediction of the model and *C* denotes the number of classes. The factor η in ab-CE motivates the model to improve the prediction on samples that are predicted with lower certainty and is increased from $\frac{1}{C}$ to a threshold of generally 0.9. This forces the model to heavily correct difficult-to-classify samples at the beginning of training.

Additionally, we compare the results using CE, ab-CE to a third loss function and focal loss (FL) [46]. Focal loss (Equation (3)) is designed to solve the class-imbalance problem in object detection and was proposed by Lin et al.; we integrate it into the CCT framework as supervised loss. When deep learning models evaluate their performance during training, the loss function calculates how many pixels were given the right object class. Since this (comparative) research paper involves the segmentation of rebar covers (i.e., small objects) and all surrounding pixels belong to the background class, the CE evaluation metric is unbalanced and in favor of the largest object class. The class imbalance in our dataset is illustrated in Table 2. Using **FL**, the average is taken of all the batches of the training samples,

(3) $$\mathrm{FL}\left(y,p\right)=-\alpha_{t}{\left(1-p_{t}\right)}^{\gamma }log\left(p_{t}\right)$$

where the modulating factor ${\left(1-p_{t}\right)}^{\gamma }$ reduces the impact of easy-to-identify samples. The focusing parameter γ is typically chosen to be 2 [46]. The parameter α is the inverse of the class frequency. This helps the model detect objects with lower presence because they will heavily impact the loss function. Since in the training dataset most pixels are labeled as background, a model can quickly over-fit on the largest class (the background) and focus on improving performance on this class only. Focal loss will prevent this.

For the unsupervised and weakly-supervised losses, we maintain the same loss functions as described by Oauli et al. For the generation of pseudo-labels, we follow their lead again and reuse the pseudo-labels generated by IRNet.

#### 4.2.3. Training Methods

**Figure 3:**
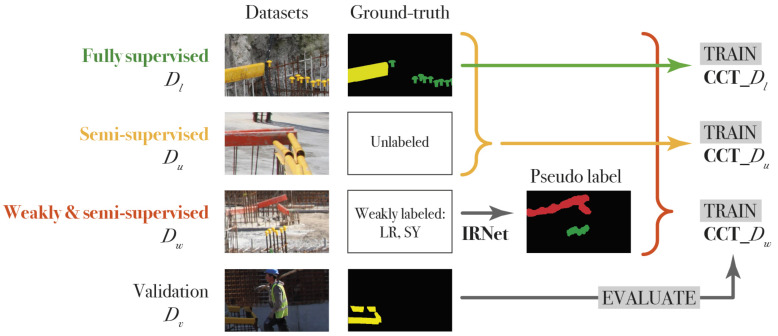
The training setup for the cross-consistency models. Four different CCT models are trained: a fully-supervised baseline CCT_$D_{l}$, a semi-supervised model CCT_$D_{u}$ and two weakly- and semi-supervised models CCT_$D_{w}$ with a regular crop size of 320×320 pixels and 304×504 pixels. All models are evaluated on $D_{v}$.

To fully evaluate CCT for construction imagery adaptation, we test all three training methods: (1) fully, (2) weakly and (3) weakly- and semi-supervised learning. First, we create a baseline using the fully-supervised dataset $D_{l}$. Second, the model is trained semi-supervised using $D_{l}$ and $D_{u}$. The training setup is illustrated in Figure 3. The labeled dataset $D_{l}$ consists of 2 images with dimensions of 3744×5616 pixels, which we analyze through a sliding window with a fixed size of 300×500 pixels. This generates 312 fully labeled images. The unlabeled dataset $D_{u}$ consists of 1064 sliding window images also of 300×500 pixels. Note that $D_{l}$ also contains sliding window images that consist of only background whereas $D_{u}$ has at least one object present in each image. Third, the CCT framework is trained weakly- and semi-supervised using the fully labeled dataset $D_{l}$, the unlabeled dataset $D_{u}$ and the weakly-labeled dataset $D_{w}$ containing 1064 images with pseudo-labels generated by IRNet.

The trained networks are validated using $D_{v}$, a validation dataset made up of 267 sliding window images each containing at least one object. Table 1 provides an overview of the datasets used in CCT compared to the data used for IRNet and Deeplabv3+. $CCT\_D_{u}$ represents the semi-supervised model and $CCT\_D_{w}$ the weakly- and semi-supervised model.

## 5. Construction Dataset

**Figure 4:**
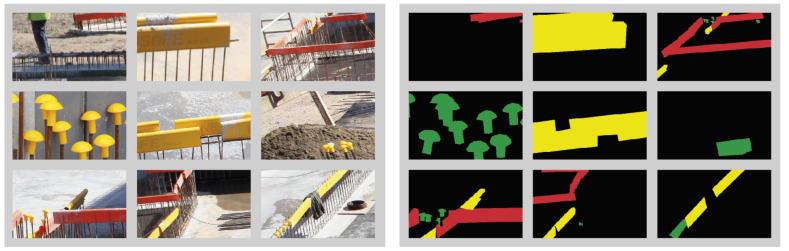
Example of training data: original input images (**left**) and ground-truth semantic labels of rebar covers (**right**).

The training images were taken at construction sites in Belgium. They were labeled at pixel level using Labelme [47]. Since the processing of images through a CNN requires a lot of memory, the images with dimension 3744×5616 pixels were subdivided into sliding windows of 300×500 pixels. The resulting 1332 images containing objects were then divided into a training set and a test set, at a ratio of 80% and 20% for the fully and weakly-supervised method. For the semi-supervised method, we used an additional 2 images of 3744×5616 pixels cut into 312 sliding windows, and we set this as the fully labeled dataset. This dataset also contains images that lack objects. The validation set for all three supervision methods was set to 267 sliding window images where each image contains at least one object. The objects that were labeled are small yellow (SY), long yellow (LY), and long red (LR) rebar covers as shown in Figure 4.

Since a construction site contains mostly other objects, the scarcity of object pixels needs to be taken into account. We do this by employing focal loss in the CCT framework, which is explained in Section 4. Table 2 shows the distribution of the object classes. Objects with a greater presence are expected to be detected more easily. As such, we expect a higher detection rate for the long yellow (LY) covers since they account for the highest pixel presence.

The construction site images are highly cluttered and have a large depth of field. The greatly varying object sizes and shadows as well as other elements obscuring the objects present a significant challenge for any network. Furthermore, since only few rebar covers are part of the scene, most pixels will belong to the background class.

## 6. Results

In this section, we describe the results of IRNet and CCT and compare them to their fully-supervised baseline models. The methods are validated on the validation dataset and intersection over union (IoU) is used as the key metric to evaluate each method’s performance. The IoU is presented as the area of true positive segmentation over the area of true positive, false positive and false negative segmentation of that object class. The IoU is thus calculated per object class: SY, LY, LR and background. The mean IoU (mIoU) shows the mean intersection over union of the four object classes. The sections below discuss the individual performance of each model and call-outs of the predictions.

### 6.1. Results of IRNet

**Figure 5:**
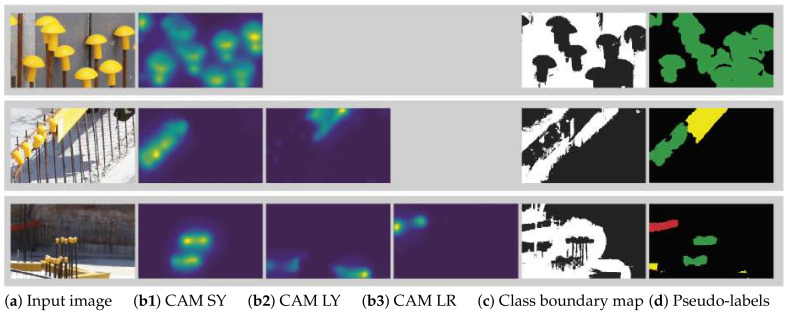
IRNet combines CAMs (**b1**–**b3**) and class boundary maps (**c**) to create the pseudo-labels (**d**) for the input images (**a**). Note that the top input image contains objects of only one and the middle image objects of only two classes, hence the different number of CAM images.

As discussed in Section 4.1, IRNet DL_fully (trained on 1065 fully labeled images), and IRNet DL_pseudo (further trained on the 1065 pseudo-labels generated by IRNet) were compared to the fully-supervised Deeplabv3+ network. Figure 5 shows the CAMs for the present object classes and class boundary maps for the input images, which were used to generate the pseudo-labels. The results are shown in Table 3. Since most of the images belong to the background class, a high IoU score is expected for that class. In terms of actual object classes, IRNet DL_fully shows the highest score for long yellow (LY) rebar covers (69.8) and the lowest score for small yellow (SY) covers (59.6). The DL_pseudo model on average scores 3 percentage points better, which is a solid increase given that this step does not require additional training data or user input. Overall, the scores are surprisingly high compared to the fully-supervised Deeplabv3+ network given the limited training data that IRNet received.

Validating IRNet on the training set, Ahn et al. achieved an mIoU of 66.5. Trained on our construction site dataset, IRNet tested on our training images achieved an mIoU of 66.7, which is similar to their result. Even though our dataset is smaller, we only have 4 object categories with clear object boundaries, which is the reason why the performance of IRNet improves. This clearly indicates that the performance of networks depends on the data and objects. However, considering the specific purpose of object detection in construction, higher quality of object boundaries might not be a priority. Rather, the rate of false positives and false negatives might be more important for construction site monitoring and building 3D models.

**Figure 6:**
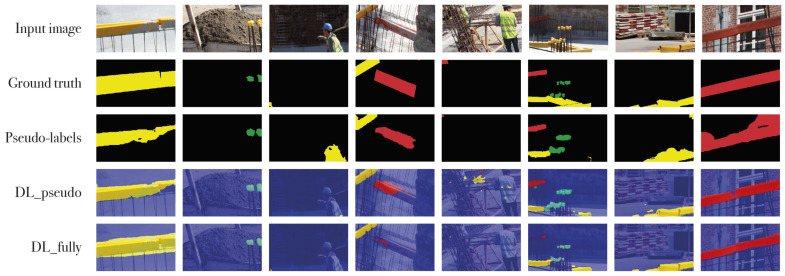
Qualitative results of IRNet. DL_pseudo is trained on the pseudo-labels generated by IRNet. DL_fully is trained on the ground truth labels.

Figure 6 shows the qualitative result of the pseudo-labels generated by IRNet and the labels generated by the Deeplabv3+ models. On average, all rebar covers are found. Furthermore, there are very few false positives especially with yellow safety vests and other yellow- and red-colored objects. This indicates that the network does not simply look for color, but also for shape. Another example of shape-based detection is the ability of the networks to differentiate between small yellow (SY) and long yellow (LY) covers.

### 6.2. Results of CCT

Prior to the classification test, several tests are performed for the CCT training. First, the encoder–decoder net is trained semi-supervised using a small labeled and a large unlabeled dataset, called CCT_$D_{u}$. A second net is trained weakly- and semi-supervised using the same two datasets but with the addition of a weakly-labeled dataset. We call this net CCT_$D_{w}$. To establish a relative evaluation, the results were compared to the fully-supervised CCT_$D_{l}$ and the fully-supervised Deeplabv3+. The former was trained using only the labeled dataset $D_{l}$. The latter is a pretrained DeepLabv3+ network which was fine-tuned on the labeled dataset.

**Figure 7:**
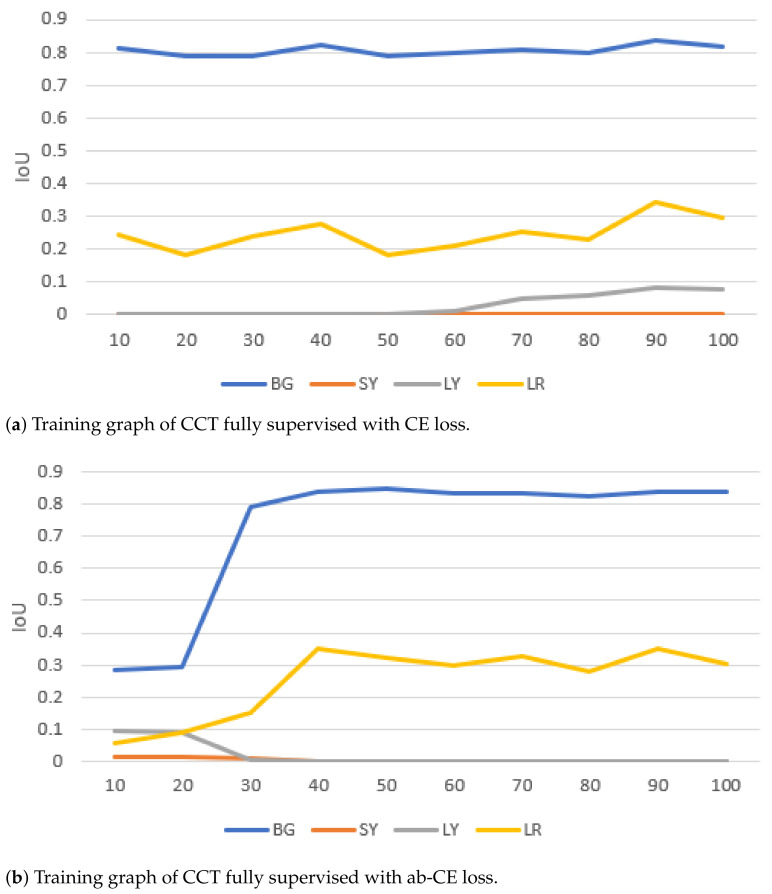

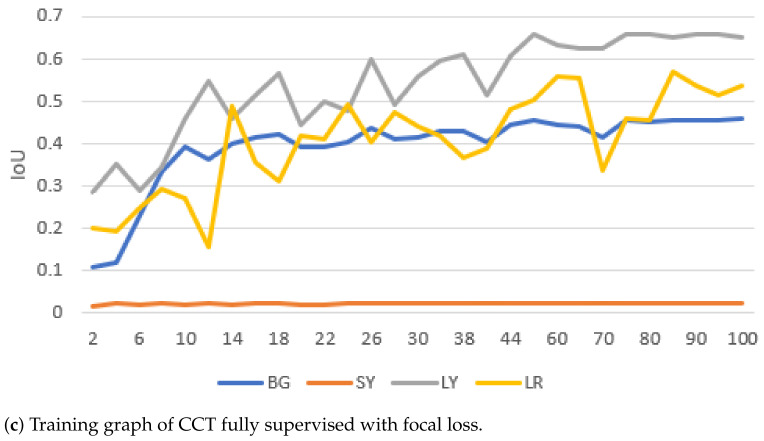
Training graphs of a fully-supervised CCT model with different loss functions. Using CE and ab-CE loss, the model focuses on the largest class and ignores the smallest class. With focal loss, the model is encouraged to also focus on smaller classes.

The CCT framework was trained using FL since CE and ab-CE loss—achieving maximum mIoU of 31.5 and 29.8, respectively—focus mostly on the background class and do not detect the two least present objects in the training images (SY and LY) (Figure 7). A learning rate of 0.001, weight decay of 0.0001 and momentum of 0.9 give the best performance over a maximum 100 epochs. The batch size is set to 3. For the other training options, we refer to [5]. Detecting the SY class remains challenging due to the small size and thus low presence of the object. Additionally, object sizes vary greatly depending on their location in the background or foreground of the image. Overall, Figure 7c shows that the focal loss function clearly outperforms the other two functions, and as such is retained for the validation of the models.

The four above models were validated on $D_{v}$ (Table 4). The improvement of the models using $D_{w}$ compared to only using $D_{u}$ is noticeable. The performance increased by almost 5 percentage points. In the paper [5], using the weakly-labeled dataset increased their model’s performance by 3.8 points, which is a slight improvement compared to the additional information provided as training material. Compared to the baseline, where CCT is only trained supervised on the small labeled dataset, there is a significant improvement exceeding the performance of Deeplabv3+.

**Figure 8:**
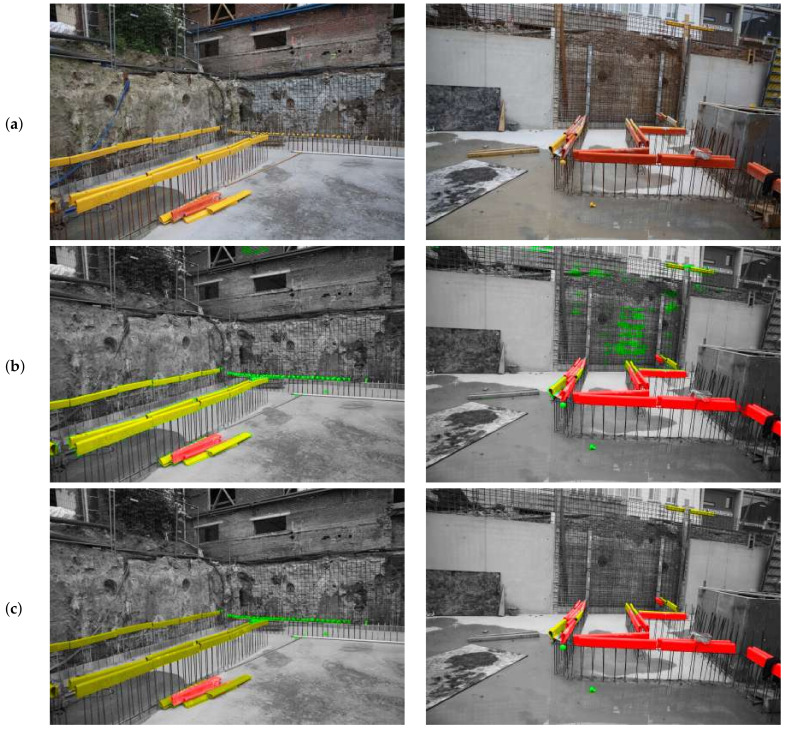
Concatenated predictions of two trained CCT frameworks on the two training images in $D_{l}$. (**a**) Input images. (**b**) Prediction of the first run of CCT_$D_{w}$ with crop size 304×504 (mIoU: 61.5 on $D_{v}$). (**c**) Prediction of the third run of CCT_$D_{w}$ with crop size 320×320 (mIoU: 69.1). All rebar covers are identified correctly. False positives occur in (**b**) in the left image on the brick wall above the excavation and on the right image in the rebar mesh against the brown earth wall.

**Figure 9:**
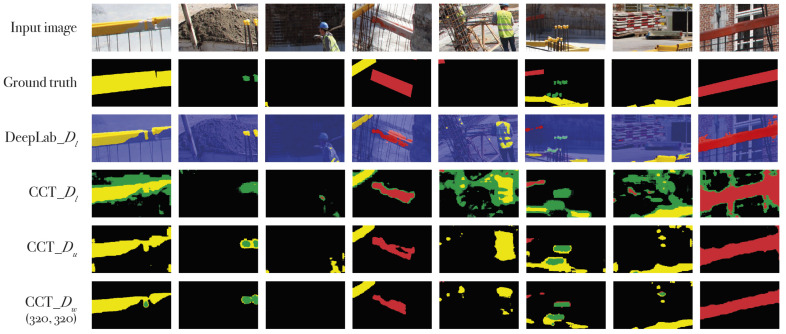
Qualitative results of the trained CCT models and DeepLabv3+ on the $D_{u}$/$D_{w}$ dataset.

The qualitative results of CCT_$D_{w}$ on the $D_{l}$ images are shown in Figure 8. The image was generated by re-concatenating the sliding windows that have been individually segmented by the network. The overlays of the segmentation over the input image show that all rebar covers were properly detected. However, there are false positives in the brick wall above the excavation and in the rebar mesh against the excavation earth wall (b). When looking closely at the long yellow covers (LY), they are visibly surrounded by pixels labeled as small yellow cover (SY). These errors are possibly caused by the color confusion between these two objects. The algorithm understands SY covers as the edges between background and LY cover. To prevent this, the training images can be enlarged (larger sliding windows) so that more of the object is visible in a single training image. In Figure 9, the results of the best performing models are compared to the Deeplabv3+ model on the unlabeled dataset $D_{u}$. The mIoU of these models is listed in Table 4.

**Figure 10:**
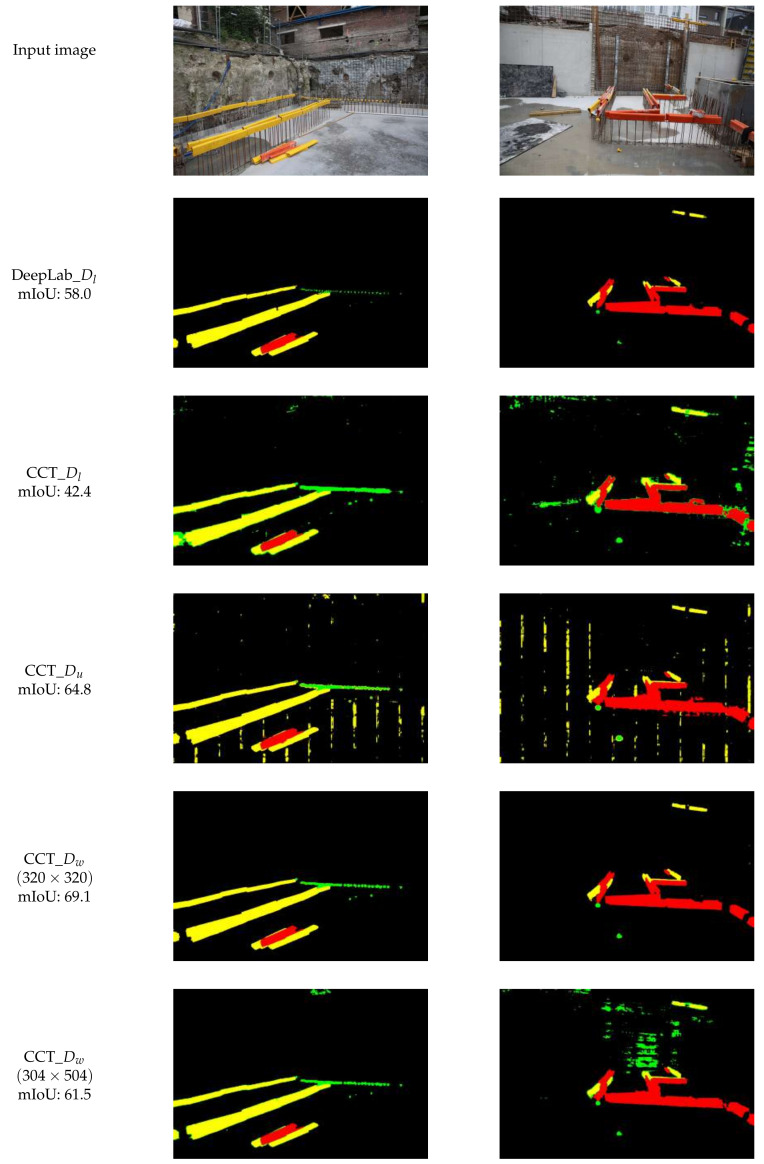
Results of trained CCT models and DeepLabv3+ on the $D_{l}$ dataset.

Figure 10 shows the predictions of the four CCT models versus Deeplabv3+ on the labeled dataset $D_{l}$. The sliding windows have been re-concatenated to generate the original large image. We purposefully show some poorly segmented cases to illustrate where failure occurs. Deeplabv3+, which is trained fully supervised on $D_{l}$, labels all rebar covers correctly without false positives. CCT_$D_{u}$ performs poorly on the SY (green) objects. The recurring vertical lines are a result of the left and right side of each sliding window being labeled as LY (even in another semi-supervised trained model the horizontal bars appear, although belonging to the SY class). CCT_$D_{w}$ with crop size 320×320 pixels performs well and classifies the objects correctly without large false positives. Increasing the crop size does not improve the segmentation and generates false positives for the SY class (green label). In comparison to analyzing each sliding window individually, looking at the final result, the performance of CCT_$D_{w}$ with crop size 320×320 pixels seems sufficient for the application of locating rebar covers.

To confirm our results, we ran multiple tests using the same settings. We trained the CCT framework with FL weakly- and semi-supervised several times. The metric results are shown in Table 5, which shows that, although the settings have not changed, the results can differ. This is due to the random initialization of the decoder network. The model does not improve when it gets stuck in a local minimum. We also adjusted the crop size for CCT_$D_{w}$ from 320×320 to 304×504 to increase performance, which is the case for the first run of CCT_$D_{w}$ but not for the second run. The weakly-supervised variant does not always outperform the semi-supervised even though weak labels are available. In addition, we note that the first run of CCT_$D_{w}$ does not detect the LY class.

## 7. Discussion

The weakly- and semi-supervised training methods discussed in this study have proven to be domain adaptable to construction site imagery. Both methods significantly reduce the need for intensive image labeling, which is the key bottleneck for deep learning adaptation. In order for construction stakeholders to decide between weakly-supervised networks such as IRNet and semi-supervised networks such as CCT, several factors need to be considered. First, there is the labeling of the training data. Although IRNet only requires image-level labels, it needs many. CCT, however, requires significantly fewer fully labeled images.

Second, the training time required to achieve maximum results while trying various settings might be of importance when the model needs to be adjusted to detect other objects or variants of the rebar covers. In our experiments, it was necessary to train CCT_$D_{w}$ four times with identical training settings (learning rate, weight decay) to obtain a model that detected all four object classes. The resulting mIoU values range from the lowest 33.8 to the highest 69.1. This is most likely caused by the model being stuck in a local minimum because further training did not improve the IoU for the object classes. Although training time of IRNet is minimal, for CCT long training times are required which are multiplied by the test runs.

Third, the performance needs to be inspected by looking at the images and not solely the metrics. Although the mIoU of CCT_$D_{w}$ attains a score of 69.1, the resulting images show many false positives and the object boundaries are ill-detected. Considering the purpose of the detection, clear and accurate boundaries might be preferred over reducing false positives or vice versa depending on the application. Although quantitatively CCT outperforms DeepLabv3+ by 11.1 percent points, it visually performs unstably providing false positives in a poor test run.

An important benefit of IRNet over semi-supervised CCT training is the shorter training time. The CAM training only requires 5 epochs and the IRNet only 3 to achieve the shown results, which comes to a total of 6 h with the GPU that was used. In comparison, the semi-supervised CCT training framework needs over 50 epochs to achieve the reported results, with one epoch lasting circa 20 min for a crop size of 320×320 pixels and circa 40 min for a crop size of 304×504 pixels. However, while only two large training images of size 3744×5616 pixels are used for the fully-supervised branch of the model, the network can still correctly identify the objects in the validation images, which are gathered from 40+ large images through a sliding window. The 267 validation images show different viewpoints and lighting conditions.

However, the results of the CCT framework in Figure 10 do not match the promising mIoU values shown in Table 5. Although CCT_$D_{w}$ with crop size 304×504 pixels achieves an mIoU of 61.5, the visual results contain too many false positives. Compared to the results of Deeplabv3+ with an mIoU of 58.0, the fully-supervised training of DeepLabv3+ with few training examples requiring less preprocessing and training time is preferable. It is thus vital to not only rely on empiric results, but also to inspect the performance visually.

In comparison to the mIoU in the initial published paper (66.5) [32], we used fewer data but generated a slightly higher mIoU (66.7) on the training data. This can be attributed to the lower number of classes and easier-to-detect objects. The image segmentations in Figure 6 show that the two DeepLabv3+ models label similarly colored objects, such as safety vests, correctly as background.

## 8. Conclusions

In this paper we discussed the adaptation of deep learning for construction site monitoring applications. More specifically, we adapted pre-existing convolutional neural networks to detect different types of rebar covers that are used to asses the positioning of wall and column elements. To deal with the problem of sparse training data, several learning strategies are considered including weakly- and semi-supervised approaches.

In the experiments, the semantic segmentation is compared for three state-of-the-art networks, i.e., DeepLab3+ (fully-supervised), IRNet (weakly-supervised) and CCT (semi-supervised). Each network is introduced to a limited training dataset to simulate a well-defined application on a construction site. Each network is optimized to yield the best results by testing different loss functions and training methods. Overall, each deep learning network is capable of yielding a satisfactory result for the rebar segmentation. The results show that while IRNet does not outperform DeepLabv3+, it does not require extensive image labeling, making it much more suited for fast adaptation. However, CCT does outperform DeepLabv3+ when using weakly-supervised and weakly- and semi-supervised learning branches, which is surprising given its limited training dataset.

Overall, we conclude that deep learning has tremendous potential to automate construction site monitoring tasks including progress estimation, quality control, quantity take-offs, safety analysis and so on. Given a relatively small set of representative images, these networks can quickly generalize for a well-defined purpose, which is currently not possible in the construction industry. As such, future work will look into detecting other objects with the semi-supervised CCT framework. Additionally, training data augmentation and zero-shot learning will be further investigated as these technologies can offer major breakthroughs in construction site monitoring automation.

## Figures and Tables

**Table 1:** Datasets of sliding window images used for training CCT, IRNet and DeepLab.

IRNet	DeepLab_$D_{l}$	CCT_$D_{u}$	CCT_$D_{w}$	Dataset
	312	312	312	fully labeled
		1064	1064	unlabeled
1064			1064	weakly labeled
267	267	267	267	validation

**Table 2:** Pixel count per object in the dataset.

Object	Pixel Count	Frequency
Small yellow covers (SY)	1,774,800	0.89%
Long yellow covers (LY)	23,307,000	11.71%
Long red covers (LR)	10,000,000	5.02%
background	164,000,000	82.38%

**Table 3:** Quantitative performance of IRNet, DL_pseudo and DL_fully. The IoU is given per class (void, SY, LY and LR).

Network	IoU (Void - SY - LY - LR)	mIoU	Training Time
IRNet DL_fully	91.7 - 59.6 - 67.3 - 69.8	72.1	4 u 58 min
IRNet DL_peudo	94.4 - 51.9 - 76.5 - 77.6	75.1	1 u 16 min
Deeplabv3+	95.5 - 58.3 - 81.3 - 81.7	79.2	<1 u

**Table 4:** Quantitative performance of the best performing CCT models trained semi-supervised using an unlabeled dataset (CCT_$D_{u}$) and a weakly-labeled dataset (CCT_$D_{w}$) compared to two baseline models (CCT_$D_{l}$ and DeepLab_$D_{l}$), evaluated on $D_{v}$.

Network	IoU (Void - SY - LY - LR)	mIoU	Training Time
CCT_$D_{u}$	89.6 - 57.4 - 61.4 - 50.9	64.8	16 u 58 min
CCT_$D_{w}$	91.8 - 58.5 - 64.8 - 61.1	69.1	36 u 37 min
CCT_$D_{l}$	45.5 - 2.10 - 65.1 - 56.8	42.4	18 u 06 min
DeepLabv3+	92.7 - 8.94 - 64.9 - 65.4	58.0	<1 u

**Table 5:** The CCT training methods yield different results over multiple runs. We ran the same tests multiple times and found that the first few epochs are deterministic for the fitting of the model. CCT_$D_{u}$ was trained on $D_{l}$ and $D_{u}$; CCT_$D_{w}$ was trained on $D_{l}$, $D_{u}$ and $D_{w}$. The models were evaluated on $D_{v}$.

Network	IoU (Void - SY - LY - LR)	mIoU	Training Time
CCT_$D_{u}$	90.0 - 43.6 - 65.9 - 42.4	60.5	14 u 32 min
	89.6 - 57.4 - 61.4 - 50.9	64.8	16 u 58 min
CCT_$D_{w}$	83.6 - 59.6 - 0.00 - 58.5	50.4	28 u 27 min
	91.8 - 58.5 - 64.8 - 61.1	69.1	36 u 37 min
(304×504)	91.9 - 29.0 - 67.2 - 58.1	61.5	32 u 48 min
	92.4 - 48.9 - 68.9 - 59.3	67.4	32 u 16 min
